# Bladder cancer immune-related markers: diagnosis, surveillance, and prognosis

**DOI:** 10.3389/fimmu.2024.1481296

**Published:** 2024-11-04

**Authors:** Tiantian Yang, Wanru Luo, Jie Yu, Huiping Zhang, Meichun Hu, Jun Tian

**Affiliations:** ^1^ College of Pharmacy, Hubei University of Science and Technology, Xianning, Hubei, China; ^2^ School of Life Science and Technology, Wuhan Polytechnic University, Wuhan, Hubei, China; ^3^ Institute of Reproduction Health Research, Tongji Medical College, Huazhong University of Science and Technology, Wuhan, Hubei, China; ^4^ Key Laboratory of Environmental Related Diseases and One Health, School of Basic Medical Sciences, Xianning Medical College, Hubei University of Science and Technology, Xianning, Hubei, China; ^5^ Department of Urology, National Cancer Center/National Clinical Research Center for Cancer/Cancer Hospital Shenzhen Hospital, Chinese Academy of Medical Sciences and Peking Union Medical College, Shenzhen, Guangdong, China

**Keywords:** bladder cancer, immune-related markers, tumor immune microenvironment, diagnosis, treatment, prognosis

## Abstract

As an immune-related tumor type, bladder cancer has been attracting much attention in the study of its markers. In recent years, researchers have made rapid progress in the study of immune-related markers for bladder cancer. Studies have shown that immune-related markers play an important role in the diagnosis, prognosis assessment and treatment of bladder cancer. In addition, the detection of immune-related markers can also be used to evaluate the efficacy of immunotherapy and predict the treatment response of patients. Therefore, in depth study of the expression of immune-related markers in bladder cancer and their application in the clinic is of great significance and is expected to provide new breakthroughs for individualized treatment of bladder cancer. Future studies will focus more on how to detect immune-related markers with low cost and high accuracy, as well as develop new immunotherapeutic strategies to bring better therapeutic outcomes to bladder cancer patients.

## Graphical Abstract

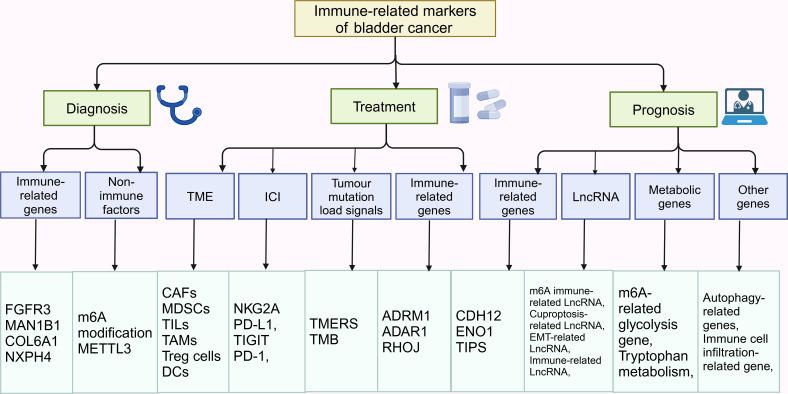

## 1 Introduction

**Figure 1:**
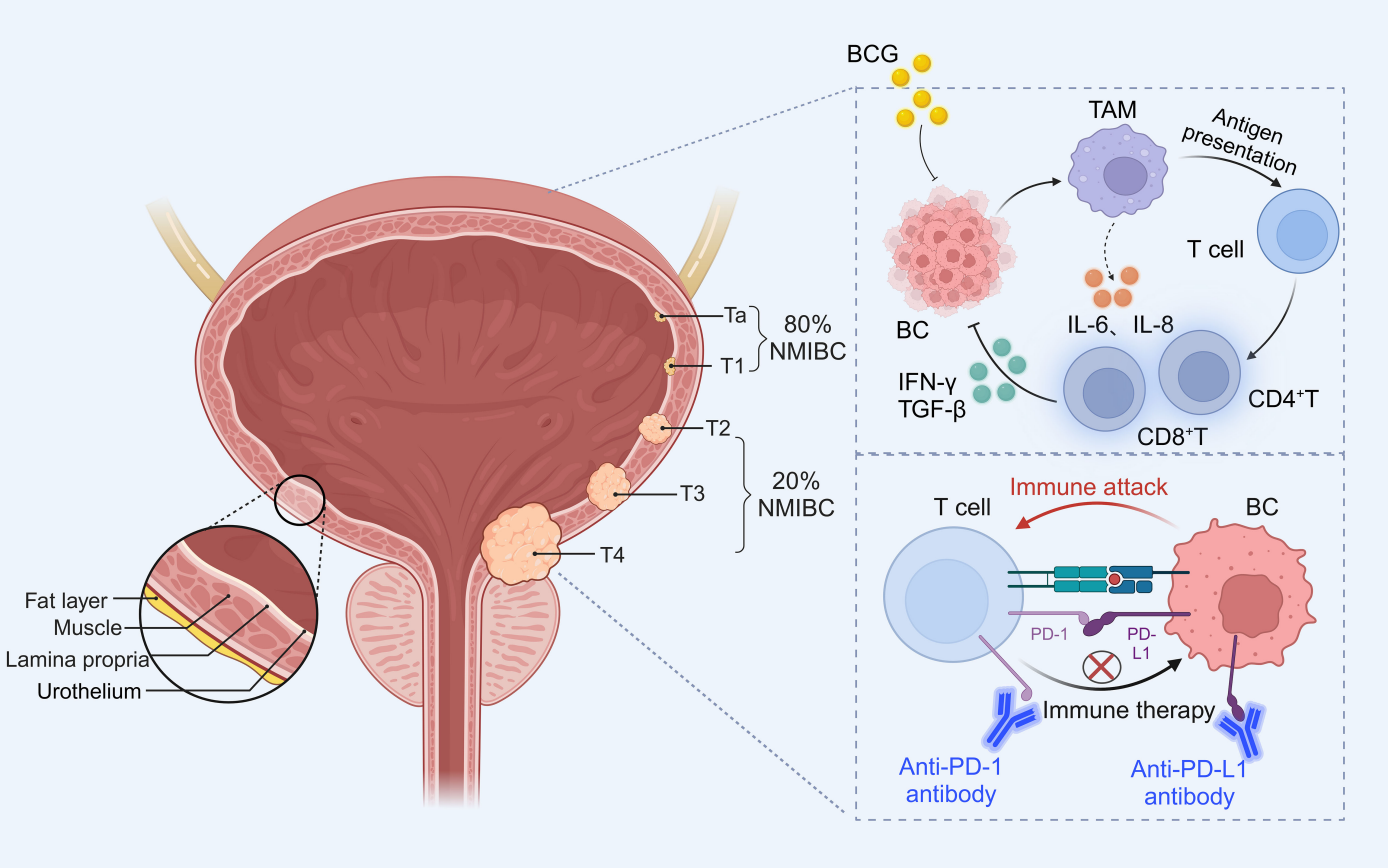
Grading of bladder cancer and occurrence of immune escape and treatment. Approximately 80% of NMIBC include Ta (invasion of mucosa), T1 (invasion of mucosal lamina propria); while T2 (invasion of muscularis propria), T3 (invasion of fatty tissue), T4 (invasion of tissues other than the bladder) belongs to the 20% of MIBC. BCG acts on immune cells in the tumor microenvironment to activate T cells to exert anti-tumor effects, while PD-1 combined with PD-L1 inhibits T cells to induce tumor cell development, and immune checkpoint inhibitors can block immune escape to inhibit bladder cancer progression.

Worldwide, bladder cancer (BC) is the tenth most common cancer and the second most common urological malignancy (1). As reported in 2020, there are more than 550,000 new diagnosed cases and more than 200,000 deaths per year (2). BC occurs mainly in the epithelial tissue of the urinary tract. Among them, non-muscle invasive bladder cancer (NMIBC) including Ta and T1 stages account for about 80% of BC. It is mainly treated by transurethral resection of bladder tumor (TURBT) followed by intravesical instillation of mitomycin C or Bacille Calmette-Guérin (BCG) to prevent recurrence. Muscle-invasive bladder cancer (MIBC) including stages T2, T3 and T4 accounts for about 20% of BC (3). The current standard of care for MIBC is radical cystectomy (RC) combined with neoadjuvant platinum-based chemotherapy (**Figure 1**).

Currently, intravesical instillation of BCG is the most common immunotherapy for NMIBC. BCG not only reacts to tumor cells by activating the immune system, leading to apoptosis, necrosis, oxidative stress etc. On the other hand, BCG can directly act on many cells in the whole tumor immune microenvironment (TME), such as macrophages, neutrophils, T cells, etc., releasing IL-6 and IL-8 cytokines to generate a cascade response to promote the host immune system to kill tumor cells (**Figure 1**). In addition to destroying the cancer cells by using the immune system, BCG also induces a high expression of PD-L1 on the surface of the tumor cells, which, in a certain way contributes to the unresponsiveness of patients to BCG (4). Even more and more studies have found that BCG treatment for NMIBC has strong cytotoxicity, and 30-40% of patients have a prognosis of progressing to MIBC. PD-L1 can provide inhibitory co-stimulatory signals to T cells after binding to the receptor PD-1, generating a negative regulatory effect, thus inhibiting the activation and proliferation of T cells, and inducing apoptosis of T cells, which mediates the occurrence of immune escape in tumor cells, avoiding the body’s immune surveillance and the development of immune response in tumor cells (5). PD-L1/PD-1 blocking drugs can disrupt the immune escape mechanism and inhibit tumor growth (**Figure 1**). Therefore, immune checkpoint inhibitors (ICIs) can be used to treat BCG non-responsive bladder cancer patients, but the overall response rate of bladder cancer patients to PD-L1/PD-1 blocking drugs is about 20-30%. The detection of PD-L1/PD-1 expression levels in tumor tissues does not fully reflect and predict the efficacy of the treatment, and the high cost and long duration of treatment with immune checkpoint inhibitors are all challenges for the wide application of immune checkpoint inhibitors in the clinic.

Traditional diagnostic and therapeutic methods for BC have certain limitations, so it has become important to find new diagnostic and therapeutic strategies. In recent years, immunotherapy has attracted widespread attention as a novel therapeutic tool. As an important component of immunotherapy, immune markers have potential diagnostic, prognostic assessment and treatment monitoring value. Immune-related markers can provide information about changes in the tumor microenvironment, which can elucidate the immune status of BC, as well as elucidate the mechanism of different immune cells in the tumor microenvironment (6). Therefore, it is hoped that immune-related markers in BC can make up for the clinical deficiencies of immunopharmacological therapy and immune checkpoint inhibitors. This review integrates various factors such as immune-related genes, TME, immune cells, tumor mutation burden (TMB), ICIs, LncRNA, and metabolic genes, providing a multi-dimensional perspective for the diagnosis, monitoring of treatment effects, and prognosis evaluation of BC. Through this comprehensive analysis, we can gain a deeper understanding of the complexity of BC and provide more precise guidance for individualized treatment of patients.

## 2 Immune-related markers in the diagnosis of bladder cancer

### 2.1 Immune-related genes

#### 2.1.1 FGFR3

FGFR3 gene mutations have been found to be highest in BC and are associated with cancer cell proliferation and migration, and tumor invasiveness (7). FGFR3 gene alterations are commonly associated with BC staging (8). FGFR3 is expressed in 49-84% of NMIBC patients and 18% of MIBC patients. In addition, FGFR3 mutations are associated with low PD-L1 expression and FGFR3 has been shown to correlate with ICIs response (9). Therefore, FGFR3 serves as a therapeutic target for bladder cancer immunotherapy and also as a predictive marker for diagnosing BC (10).

#### 2.1.2 MAN1B1

The study demonstrated higher MAN1B1 expression in bladder cancer patients than in normal tissues from The Cancer Genome Atlas (TCGA) and Genotype Tissue Expression (GTEx) databases. Survival analysis showed that patients with high MAN1B1 expression had significantly worse overall survival (OS), disease-specific survival (DSS) and progression-free interval (PFI). The abundance of acquired immune cells (helper T cells and NK cells) was found to be negatively correlated with MAN1B1 expression by single-sample gene set enrichment analysis (ssGSEA), whereas the abundance of innate immune cells (Th2 cells, macrophages, Th1 cells, neutrophils) was positively correlated with MAN1B1 expression (11). Multifactorial analysis also confirmed that MAN1B1 expression was an independent prognostic factor for OS in bladder cancer patients. Thus, MAN1B1 is not only a novel biomarker for the diagnosis of BC, but also for assessing the prognosis and the degree of immune infiltration in bladder cancer patients (12).

#### 2.1.3 COL6A1

In recent years, it has been found that there is a significant effect of TME on the growth and spread of tumor cells, in which extracellular matrix (ECM) is an important component of TME, regulating the growth, invasion, apoptosis, drug resistance and metastasis of tumor cell (13). Human collagen VI (COL6A1) is among the most predominant ECM proteins and is usually involved in tumor cell growth and related metastasis (14). Bioinformatics analysis screened COL6A1 as a key hub gene for the diagnosis of BC and verified the correlation between COL6A1 expression and BC in the cohort, and COL6A1 is an important prognostic risk-associated gene for BC, which is closely related to the malignant progression and prognosis of BC. In addition, COL6A1 expression level was closely associated with immunotherapy in patients with advanced bladder cancer, and bladder cancer patients with high COL6A1 expression showed poorer responsiveness and efficacy to immunotherapy. In conclusion, COL6A1 is closely related to the malignant progression and prognosis of bladder cancer patients and the efficacy of immunotherapy, and it is a promising biomarker (15).

#### 2.1.4 NXPH4

Neurexophilin 4 (NXPH4) is a synaptic secretory protein belonging to the NXPH family. NXPH1 and NXPH2 have been reported to be highly expressed in pancreatic cancer (16). Sun et al. found that the expression of NXPH4 in BC was significantly higher than that in normal tissues. Survival analyses confirmed that NXPH4 was closely associated with OS and PFS in bladder cancer patients, and positively correlated with poor prognosis. NXPH4 was determined to be an independent prognostic factor for BC using univariate and multivariate cox regression analysis. In addition, memory B cells, M0 macrophages and resting dendritic cells were found to be increased in the high NXPH4 expression group. The immunity score and stromal score were higher in the low NXPH4 expression group than in the high NXPH4 expression group. The low NXPH4 expression group had a higher positive response to immunotherapy compared with the high NXPH4 expression group. In conclusion, NXPH4 not only assesses the prognosis of BC, but also facilitates the diagnosis of BC (17).

### 2.2 Non-immune factors

Genetic and epigenetic alterations in DNA, such as mutations and copy number variation (CNV), are frequently reported in BC. N6-methyladenosine (m6A) modification is one of the most common RNA modifications in mammalian systems. It is dynamically regulated by methyltransferases (METTL3, METTL14, METTL16), binding proteins and demethylases. Among them, METTL3 promotes increased growth and progression of BC (18). CNVs of METTL3, METTL14 and METTL16 were found to correlate with the molecular characteristics of bladder cancer patients. CNVs of METTL3 were associated with OS of bladder cancer patients. METTL3 may affect the prognosis of BC by regulating the level of immune infiltration, suggesting that METTL3 is an immune-related biomarker for bladder cancer and may become an indicator for early diagnosis in the future (19).

Immune-related genes (such as FGFR3, MAN1B1, COL6A1, NXPH4) and other factors (METTL3) play important roles in the diagnosis of BC (**Table 1**). Immune-related genes influence the occurrence and development of tumors by regulating the proliferation, migration, and invasion of tumor cells. Mutations in the FGFR3 gene are associated with low PD-L1 expression in BC, high expression of MAN1B1 is related to poor prognosis, high expression of COL6A1 in tumor cells promotes tumor growth and metastasis, and high expression of NXPH4 is associated with poor prognosis in BC. METTL3, as a methyltransferase, affects the proliferation and progression of bladder cancer cells by regulating the m6A modification of RNA. By detecting these biomarkers, BC can be diagnosed more accurately, and personalized treatment plans can be provided for patients.

**Table 1:** Summary of the characteristics of different immune-related markers for the diagnosis of bladder cancer.

Markers		Characteristics	References
Immune-related genes	FGFR3	FGFR3 mutations are associated with low PD-L1 expression.	(9)
	MAN1B1	MAN1B1 is negatively associated with acquired immune cells and positively associated with innate immune cells	(11)
	COL6A1	COL6A1 is the predominant ECM protein and is usually involved in tumor cell growth and metastasis	(14)
	NXPH4	NXPH4 is a synaptic secretory protein, highly expressed in BC	(17)
Non-immune factors	METTL3	METTL3 promotes increased growth and progression of BC	(18)

## 3 Immune-related markers in the treatment of bladder cancer

### 3.1 Tumor immune microenvironment

**Figure 2:**
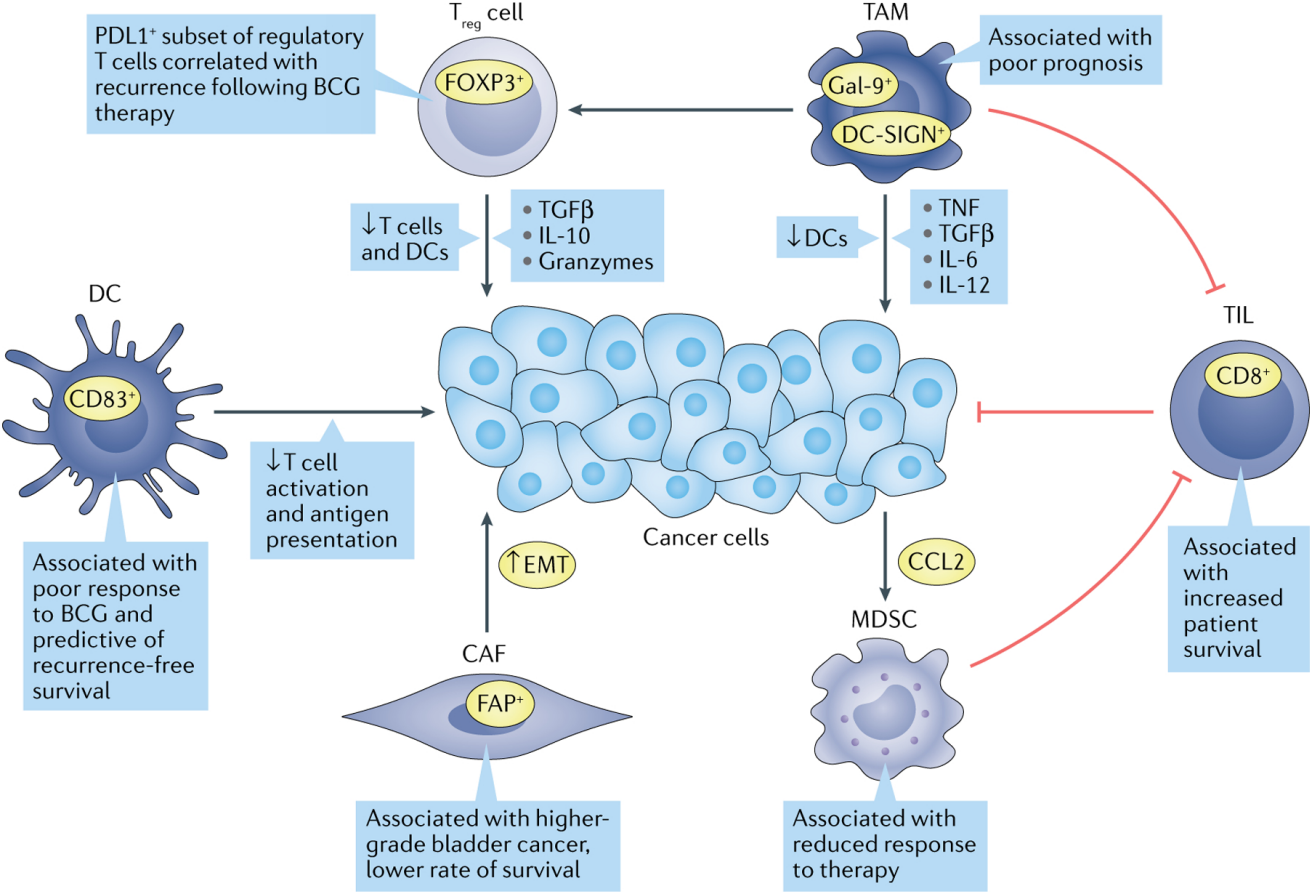
Immune cell types are associated with bladder cancer progression. Many tumor-infiltrating immune cells correlate with treatment response and survival outcomes in bladder cancer patients. Green arrows indicate oncogenic associations, while red indicates anti-tumor activity (3).

The success of immunotherapy is largely dependent on TME, and dysregulation of TME promotes malignant progression from NMIBC to MIBC. Some studies have reported higher proportions of macrophages, memory-activated CD4^+^ T cells and activated natural killer cells (NK) and lower proportions of resting memory CD4^+^ T cells in MIBC compared to NMIBC. Studies have shown that tumor infiltrating cells are more infiltrated in MIBC than in NMIBC. NK cells play a crucial role in the early immune response to tumors (20, 21). Elevated levels of tumor-infiltrating neutrophils correlated significantly with tumor stage (22). The main antigens of tumor-associated macrophages are CD68 and CD163, and the proportion of CD68^+^ cells correlates with stage. The level of B cells is higher in BC than in normal tissues whereas the expression of PD-1 is lower in high-risk NMIBC, and significantly higher on CD4^+^ and CD8^+^ T cells (23, 24). Ectopic tertiary lymphoid structure (TLS) may be associated with disease aggressiveness, and TLS was detected in both NMIBC and MIBC. In conclusion, massive immune cell infiltration was associated with OS and PFI, and changes in tumor-infiltrating immune cells depended on the proportion of CD8^+^ T cells, activation memory CD4^+^ T cells, and the proportion of T helper cells (**Figure 2**). Patients with higher CD4^+^ T cell infiltration had significantly shorter OS than those with lower infiltration (25). Taken together, these infiltrating immune cell types are important in the treatment of BC, and TME and immune cell infiltration show promise as predictors of bladder cancer progression and response to treatment (26).

#### 3.1.1 Cancer-associated fibroblasts (CAFs)

TME includes not only immune cells but also stromal cells, and CAFs are the main stromal cell type in the TME. CAFs are involved in ECM remodeling by secreting cytokines that interact with tumor and immune cells, and CAFs promote tumor angiogenesis and metastasis to influence tumor progression and ultimately the course of the immune response (27). CAFs are significantly increased in bladder cancer patients compared to normal bladder tissue (28). The expression of CD90 and FAP, markers of CAFs, was found to be positively correlated with the invasiveness of BC, and the expression of FAP was positively correlated with the invasiveness and negatively correlated with the survival of bladder cancer patients (29, 30). In addition, the expression of CAF markers was closely correlated with the expression of epithelial-mesenchymal transition (EMT) markers in cancer cells, and stimulated fibroblasts could induce EMT in cancer cells *in vitro* (31).

#### 3.1.2 Marrow derived sexual inhibition cells (MDSCs)

In normal tissues, MDSCs limit excessive inflammation and prevent autoimmune diseases (32). In cancer, MDSCs promote immune evasion, stimulate proliferation, facilitate invasion and metastasis, and ultimately hinder immunotherapy (33). In bladder cancer patients, MDSCs are enriched in cancer tissue and inhibit CD8^+^ T cell activity. It has been shown that gemcitabine induces bladder cancer cells to secrete CCL2, which promotes MDSC recruitment and limits response to treatment (34).

#### 3.1.3 Tumor-infiltrating lymphocytes (TILs)

TILs indicate cell-mediated host responses to tumor cells. Recognition of exogenous antigens by CD8^+^ T cells can induce apoptosis by releasing cytotoxins or death ligands (35). In MIBC, TILs localized in the tumor-adjacent stroma have been shown to predict bladder cancer immunophenotype, patient survival and molecular tumor subtype. Tumors are usually classified according to the degree of TIL presence: uninflamed tumors with low TIL infiltration, inflamed tumors with low or high TIL infiltration, and moderately TIL-infiltrated tumors but lacking PDL1^+^ immune cells. High inflammation in tumors has been shown to be associated with increased survival in almost all cancer patients (3).

#### 3.1.4 Tumor-associated macrophages (TAMs)

TAMs promote cancer growth and metastasis by secreting growth factors, cytokines, and proteases, and they also promote chronic inflammation by releasing mediators such as TNF, TGF-β, IL-6, and IL-12. Macrophages are usually differentiated into “M1” or “M2” subtypes, with the M1 type promoting an acute inflammatory response in tumor cells and the M2 type promoting chronic inflammation, which in turn leads to immunosuppression and promotes tumor growth (36). TAMs are predominantly of type M2 in patients with bladder cancer and are found in greater numbers in high-grade disease compared to low-grade disease (37). In MIBC, the frequency of Gal-9^+^ TAMs was positively correlated with tumor stage (38). Another subclass of M2 macrophages, DC-SIGN^+^ TAMs was also associated with a poorer prognosis (39).

#### 3.1.5 Regulator T (Treg)

Stromal cells recruit M2 macrophages into the TME and promote Treg cell infiltration (40). Treg cells secrete inhibitory cytokines, granzyme A and granzyme B to inhibit T cell activation and proliferation and regulate DCs maturation and function (41). Urine analysis of patients with NMIBC showed that Treg cells were essentially absent before BCG injection, but persisted after treatment. Moreover, high levels of Treg cells in urine were associated with rapid relapse after BCG treatment. In MIBC, the ratio of CD8^+^ T cells to Treg cells correlated with response to neoadjuvant chemotherapy, with patients with a ratio of less than 1 not responding (42).

#### 3.1.6 Dendritic cells (DCs)

DCs are antigen-presenting cells that recognize pathogens and danger-associated signals, deliver them to the initial T cells and contribute to the anti-tumor immune response. Dysfunction of DCs in the TME leads to ineffective antigen presentation and immune escape (33). In NMIBC, high levels of tumor-infiltrating dendritic cells correlate with poor response to BCG and predict relapse-free survival (43). The ratio of CD8^+^ T cells to various immunosuppressive cell types has been found to be a prognostic marker for BC, and in NMIBC, a ratio of CD8^+^ T cells to MDSC of less than 1 predicts shorter RFS (42).

### 3.2 Immune checkpoint inhibitors

#### 3.2.1 NKG2A and PD-L1

Studies have shown that NKG2A is a PD-1/PD-L1 axis-associated immune-dependent factor, and NKG2A is frequently co-expressed with PD-1 in bladder cancer patients. Patients with NKG2A and PD-L1 have significant treatment efficacy with cisplatin-based adjuvant chemotherapy and PD-L1 inhibitors. Therefore, NKG2A and PD-L1 were used as combined biomarkers to predict the treatment response of MIBC to adjuvant chemotherapy (ACT) and PD-L1 blockade. The results of the study demonstrated a positive correlation between NKG2A expression and PD-L1 expression in the IMvigor210 cohort, and NKG2A and PD-L1 expression were significantly associated with OS. It was shown that the NKG2A and PD-L1 expression group was positively correlated with more immune cell infiltration (CD8^+^ T cells, B cells, M1 macrophages, and NK cells) and inflammatory factors (IFN-γ, GZMB, and PRF-1), which implies that NKG2A and PD-L1 expression is not only associated with the tumor microenvironment but also with the inflammatory microenvironment. Therefore, NKG2A and PD-L1 were used as combined biomarkers to predict the therapeutic response of MIBC to ACT and PD-L1 blockade (44).

#### 3.2.2 TIGIT and PD-1

Previous studies have shown that TIGIT occurs predominantly on Treg cells and depleted T cells and less frequently on effector T cells (45). High PD-1 expression was detected in both depleted T cells and effector T cells (46). Recent studies have found an association between the level of TIGIT or PD-1 expression and survival in MIBC, with TIGIT and PD-1 expression affecting the functional status of TME and CD8^+^ T cells. Dual checkpoint blockade of TIGIT and PD-1 enhances anti-tumor immunity and patient survival (47, 48). Findings suggest that low TIGIT low PD-1 class I patients have low infiltration of immune cells, have high FGFR3 mutations and are sensitive to FGFR3 targeted therapy. Class II patients with low TIGIT high PD-1 had a high infiltration of immune cells with increased CD8^+^ T cells and had the best prognosis. High TIGIT high PD-1 class III patients can be immunosuppressed by suppressing CD8^+^ T cells (49). These patients have the worst survival but can benefit more from adjuvant chemotherapy and anti-PD-L1 immunotherapy. In conclusion, TIGIT/PD-1 stratification can be used as a predictor of clinical prognosis. In addition, a novel immune classification based on TIGIT and PD-1 may optimize the systemic treatment of patients with ACT and PD-L1 inhibitors, which would point to a new path for future clinical practice (50).

### 3.3 Tumor mutation load signals

Studies have shown that tumor mutation load in MIBC patients is negatively correlated with prognosis. TME related signals (TMERS) were found to be an independent prognostic factor in MIBC, and TMERS was positively correlated with immune infiltration, expression of immune checkpoints and molecular subtypes. The results showed that the number of immunosuppressive cells, macrophages, and Tregs was significantly increased in the high TMERS group, and produced IL-10 and TGF-β, and directly or indirectly inhibited the activation of T cells and NK cells within the TME, suppressing the immune response and promoting the tumorigenesis. The high TMERS group mainly clustered in basal/scc-like molecular subtypes, which mainly inhibited immune cell infiltration and activated the EMT/TGF-β pathway and up-regulated immune checkpoints. TMERS is a potent biomarker for predicting response to immunotherapy. Tumor mutation burden (TMB) can also be used as a predictive biomarker for immunotherapy, and TMB has been shown to be a prognostic factor with the potential to predict response to ICIs (51, 52). The combination of TMERS and TMB allows for clear and accurate MIBC patient stratification, with patients with high TMB/low TMERS having the best prognosis and those with low TMB/high TMERS having the worst prognosis. In addition, immunotherapy response rates were highest in the high TMB/low TMERS group and lowest in the low TMB/high TMERS group. Taken together, TMERS and TMB combination is a strong independent prognostic factor and predictive biomarker of MIBC response to ICIs (53).

### 3.4 Immune-related genes

#### 3.4.1 ADRM1

Deubiquitinated proteins are considered as key oncogenes or tumor suppressor genes in various malignancies (54). ADRM1 is a ubiquitin receptor located on the 26S proteasome, which plays a role in cell adhesion, deubiquitination and protein hydrolysis. ADRM1 was found to be significantly overexpressed in BC. In bladder cancer patients, high expression of ADRM1 was associated with worse OS. Immune-related pathways were found to be highly correlated with ADRM1 high expression group. In the ADRM1 high expression group, the expression of PD-L1 and PD-1 was increased, and patients with high ADRM1 expression were more likely to benefit from anti-PD-L1 therapy, with significantly higher proportions of CD8^+^ T cells and M1 macrophages, and significantly lower proportions of CD4^+^ T memory cells and mast cells, and higher TMB scores with a higher stemness index. The ADRM1 high expression and low expression groups of the Chemotherapy efficiency results revealed that patients in the ADRM1 low expression group were more sensitive to multiple chemotherapeutic agents. Therefore, ADRM1 may be a valuable biomarker for bladder cancer patients. Patients may choose immunotherapy or chemotherapy as the best treatment option based on ADRM1 level (55).

#### 3.4.2 ADAR1

Aberrant expression and dysfunction of ADAR1 may have tumorigenic or tumor-suppressive effects, affecting tumor proliferation, invasion and response to immunotherapy (56). It was found that the expression of ADAR1 in BC was significantly higher than that in normal tissues, and high expression of ADAR1 predicted a higher pathological grade and a worse prognosis. Moreover, ADAR1 was found to be associated with infiltrating immune cells in BC, and with the activation of M1 macrophages, with significantly higher levels of infiltration of immune cells such as CD4^+^ T cells, macrophages and DCs, which were positively correlated with the expression of several immune checkpoints, and the high expression of ADAR1 predicted a better response of BC to PD-1 blockade, which may indicate a good response to immunotherapy. In addition, *in vitro* experiments showed that ADAR could effectively promote the proliferation, migration and invasion of BC. Taken together, ADAR plays an important role in the occurrence, progression and immunotherapy of BC, and it can be used as a predictive marker and therapeutic target for immunotherapy response (57).

#### 3.4.3 RHOJ

RHOJ regulates the cytoskeleton, which promotes the metastatic potential of cancer cell (58–60). Moreover, RHOJ is involved in neovascularisation, and tumor angiogenesis is a key condition for tumor development (61, 62). The findings suggest an association between elevated RHOJ expression and poor prognosis and reduced survival, suggesting its potential value as a prognostic indicator. A clear association between RHOJ gene expression and tumor stage was also found. Therefore, the higher the RHOJ expression, the higher the degree of malignancy of the tumor. High RHOJ expressing bladder cancer may exhibit enhanced immune responses in the TME, possibly associated with an increased monocyte-to-lymphocyte ratio. There was a significant positive correlation between RHOJ expression and both Treg cells and T cell depletion. Thus, RHOJ may play a role in regulating the tumor-immune system interactions, and thus may contribute to immune evasion. In conclusion, RHOJ is a key player in disease progression and immune regulation and a possible therapeutic target (63).

In the treatment of BC, CAFs, MDSCs, TILs, TAMs, Tregs, and DCs cells regulate the TME by secreting various factors, affecting the progression of the tumor and its response to treatment. They can also influence the efficacy of ICIs by suppressing or promoting immune responses. TMB is an important biomarker for predicting the response to immunotherapy, with high TMB tumors potentially having more neoantigens, which can more effectively activate the immune system, thereby enhancing the efficacy of ICIs. ICIs block inhibitory signals, restoring the anti-tumor activity of T cells. Immune-related genes influence the patient’s response to immunotherapy by regulating the activity and infiltration of immune cells in the TME. They collectively shape the TME, affecting the efficacy of immunotherapy (**Table 2**). By comprehensively analyzing these factors, the treatment response and prognosis of bladder cancer patients can be better predicted, providing a basis for personalized treatment.

**Table 2:** Summary of the characteristics of different immune-related markers for the treatment of bladder cancer.

Markers		Characteristics	References
Tumor immune microenvironment	CAFs	CAFs are involved in ECM remodeling and CAFs promote tumor progression affecting the immune response	(27)
	MDSCs	MDSC inhibits CD8^+^ T cell activity	(33)
	TILs	TILs release cytotoxins to induce apoptosis	(35)
	TAMs	M1-TAMs promote acute inflammatory responses, whereas M2-TAMs promote chronic inflammation	(36)
	Treg cells	Treg cells produce inhibitory cytokines to suppress T cells, DCs	(41)
	DCs	DCs recognize danger-associated signals delivered to initial T cells to exert anti-tumor immune responses	(33)
Immune checkpoint inhibitors	NKG2A, PD-L1	NKG2A positively correlates with PD-L1	(44)
	TIGIT, PD-1	Classifying bladder cancer patients with TIGIT and PD-1	(50)
Tumor mutation load signals	TIMERS	TMERS predicts immunotherapy	(53)
	TMB	TMB predicts ICI response	(52)
Immune-related genes	ADRM1	Immune pathway is highly correlated with ADRM1	(55)
	ADAR1	ADAR1 is associated with infiltrating immune cells	(57)
	RHOJ	RHOJ expression is positively correlated with T-cell exhaustion	(63)

## 4 Immune-related markers in the prognosis of bladder cancer

### 4.1 Immune-related genes

#### 4.1.1 CDH12

Tumor cells were found to express Cadherin 12 (CDH12), which was predominantly found in the basal/squamous, luminal-infiltrating and neuroendocrine-like subtypes, and was significantly absent in the luminal papillary and luminal indeterminate subtypes. It was also found that patients with CDH12-enriched bladder cancer had a poorer postoperative prognosis but a better prognosis with ICT. CDH12-enriched cells expressed PD-L1 and PD-L2, which may contribute to the effectiveness of BC for ICT through CD49a-mediated co-localization with depleted T cells. In conclusion, CDH12 enrichment stratified patients as a better predictor of prognosis than current bladder cancer subtypes. CDH12 scores predicted poor prognosis in patients with MIBC in addition to poor response to neoadjuvant chemotherapy, and response to immunotherapy after chemotherapy (64).

#### 4.1.2 ENO1

The mRNA expression of ENO1 was found to be upregulated in BC by database. Using Kaplan-Meier survival analysis, it was found that the higher the ENO1 expression, the worse the prognosis. Combining ENO1 expression with clinicopathological factors to construct a nomogram model had a good prognostic effect, with an AUC value of 0.762. Analysis using the CIBERSORT algorithm revealed that there were eight types of immune cells with significant differences in ENO1 expression. ENO1 expression was associated with activated memory CD4 cells, resting NK cells, M0 macrophages, neutrophils, infiltration of Treg cells was significantly correlated. Taken together, these findings suggest that ENO1 may play an important regulatory role in tumor immunity and that ENO1 may serve as a promising prognostic biomarker for predicting prognosis associated with the TME (65).

#### 4.1.3 TP53-associated immune prognostic signature (TIPS)

Mutations in the TP53 gene, one of the most commonly mutated genes in human cancers, are associated with progression and prognosis in BC (66). The TP53 gene is the most commonly mutated type in MIBC (67). Bladder cancer patients with TP53 mutations have a poorer prognosis compared to patients without TP53 mutation (68, 69). TP53 mutations were found to be strongly associated with immune-related biological processes and three TIPS genes (ORM1, PTHLH and CTSE) were screened to predict OS and immunotherapy response in MIBC. ORM1 and PTHLH are associated with tumor immunity and correlate with immune signaling pathways. CTSE is involved in antigen processing and maturation of secreted proteins, and can regulate MHC class II-mediated processing of antigenic peptides during antigen presentation. Therefore, TIPS served as an independent prognostic factor for predicting OS in MIBC, with AUCs of 0.625, 0.643, and 0.640 for 1, 3, and 5 year predicted OS for TIPS, respectively. It was also found that the poor prognosis of the high-risk group may be related to a high degree of immunosuppression and low immune responsiveness, which promotes tumor recurrence and metastasis. Therefore, the high-risk prognostic group may be more sensitive to anti-PD-1 therapy. TP53 mutation-based TIPS is a potential prognostic feature or therapeutic target for MIBC, laying the foundation for new therapeutic strategies for MIBC (70).

### 4.2 Long chain non-coding RNA(LncRNA)

#### 4.2.1 m6A immune-related LncRNA

It has been shown that m6A-LncRNAs play a key role in tumourigenesis and innate immunity (71). A risk model containing 11 LncRNAs was built from m6A-LncRNA and immune-related LncRNA, which was confirmed by ROC and Kaplan-Meier analysis to predict the prognosis of bladder cancer patients. Its model risk score was significantly correlated with tumor malignancy or immune cell infiltration. Meanwhile, there were significant differences between the low-risk and high-risk groups in terms of tumor mutational burden and stemness scores. Patients in the low-risk group had a higher mutational burden, and the immunophenotype score was greater in the low-risk group than in the high-risk group, suggesting that patients in the low-risk group may be more immunogenic and may be more sensitive to immunotherapy. Infiltrating immune cells CD4^+^ and CD8^+^ T cells, DCs and macrophages were more abundant in the high-risk group than in the low-risk group. And the stemness index was higher in the low-risk group, so this study established a m6A immune-related LncRNA risk model, which can be used to predict the prognosis of BC, and response to immunotherapy (72).

#### 4.2.2 Cuproptosis-related LncRNA

Cuproptosis is a copper-triggered programmed cell death associated with the development, prognosis and immune response of various cancers (73). LncRNAs can serve as prognostic biomarkers and are involved in the progression of BC (74). A prognostic signature of cuproptosis-related LncRNAs in BC was constructed, including 8 LncRNAs (RNF139-AS1, LINC00996, NR2F2-AS1, AL590428.1, SEC24B-AS1, AC006566.1, UBE2Q1-AS1, and AL021978.1) to build a predictive model, its risk scores with AUCs of 0.692, 0.692 and 0.721 at 1, 3 and 5 years, respectively. Cuproptosis-related LncRNA prognostic features had better predictive ability compared with other clinicopathological features. Patients in the high-risk group were positively associated with tumor-infiltrating immune cells and negatively associated with memory B cells, plasma B cells, B cells, CD4^+^ T cells, Treg cells and T cells. Bladder cancer patients with low-risk scores have higher TMB levels, and bladder cancer patients with high-risk scores may be more likely to be resistant to chemotherapy and immunotherapy and have poorer survival. Thus, cuproptosis-related LncRNA could help predict the prognosis and immune outlook of BC (75).

#### 4.2.3 EMT-related LncRNA

EMT is the phenomenon of epithelial to mesenchymal cell transformation, which is a process of cell de-differentiation or re-differentiation (76). Through the process of EMT, the migration and motility of cancer cells are enhanced, promoting invasion and metastasis (77). Epithelial tumors account for more than 95% of the pathological types of BC, so EMT may influence the development and progression of BC (78). LncRNA regulates cancer cell growth and progression and are potential biomarkers for predicting cancer risk and survival outcomes (79, 80). Fourteen EMT-related LncRNA were found to be independent prognostic factors in bladder cancer patients. EMT-related LncRNA features predicted OS, in which the AUC values were all greater than 0.73. The prediction model of EMT-related LncRNA prognostic features in combination with clinicopathological features was able to accurately predict the OS of bladder cancer patients, which has great potential for clinical applications, including individualized prognosis and treatment. Thus, the EMT-related LncRNA features are essential for the diagnosis of BC and can predict the prognosis and progression of bladder cancer patients (81).

#### 4.2.4 Immune-related LncRNA

In recent years, LncRNA has been found to be associated with tumor progression and immune microenvironment (82–84). Immune-related prognostic LncRNA signature (IRPLS) was constructed from four LncRNA containing RP11- 89, PSORS1C3, LINC02672, and MIR100HG. IRPLS predicted OS with good accuracy, with AUC of 0.653, 0.656, and 0.684 at 1, 3, and 5 years, respectively. Immune cell distribution showed a significant difference between high-risk and low-risk groups. The proportion of T cells was significantly higher in the low-risk group than in the high-risk group. The interstitial and immunological scores were higher in the high-risk group compared to the low-risk group. In addition, a series of *in vitro* experiments revealed that RP11- 89 was able to promote cell proliferation and invasive ability in BC, and at the same time, RP11- 89 targeted miR- 27a- 3p and up-regulated the expression of PPARγ in BC to inhibit apoptosis. IRPLS significantly predicted poor clinical outcomes and immune microenvironmental deficits in patients with bladder cancer, which may provide a new individualized immune therapy target (85).

Wu et al. identified 8 LncRNAs associated with bladder cancer prognosis and immunotherapy response (MIR181A2HG, AC114730.3, LINC00892, PTPRD-AS1, LINC01013, MRPL23-AS1, LINC01395, AC002454.1). In addition, 8 LncRNAs were associated with RFS in BC. This profile was also associated with immune cell infiltration (macrophages M0, M2, Tregs, CD8^+^ T cells and neutrophils) and ICI and immunotherapy-related biomarkers. Thus, 8 LncRNAs can predict bladder cancer prognosis and immunotherapy response (86). Wang et al. identified 7 immune-related LncRNAs with prognostic value in BC (Z84484.1, AC009120.2, AL450384.2, AC024060.1, TNFRSF14-AS1, AL354919.2, OCIAD1-AS1). 7 immune-related LncRNAs with an AUC value of 0.734, demonstrating that the characteristics of the 7 immune-related LncRNAs were an independent prognostic factor. Immune status differed between the low and high-risk groups, with lower tumor purity and higher stromal components in the high-risk group compared to the low-risk group. CD8^+^ T cells, DCs and macrophages were associated with the immune-related LncRNA features. In conclusion, seven immune-associated LncRNAs can be used as prognostic markers for BC (87).

### 4.3 Metabolic genes

#### 4.3.1 m6A-related glycolysis gene

m6A is the most common mRNA and non-coding RNA modification in eukaryotic cell, and its aberrant regulation is associated with glycolysis (88, 89). Thirty-four m6A glycolytic genes associated with bladder cancer were screened. 1, 3 and 5 year AUCs of the 34 crossover genes were greater than 0.6, suggesting that the survival risk model can be effectively used as a prognostic model. In addition, the expression of IP6K2 and PLA2G2F was significantly negatively correlated with disease risk. It was demonstrated by *in vitro* experiments that IP6K2 and PLA2G2F knockdown promoted bladder cancer cell proliferation and colony formation. IP6K2 expression was found to be positively correlated with M2 macrophage, MHC class 1 and NK cell function, and PLA2G2F expression was negatively correlated with activated DCs. Risk scores were positively correlated with activated memory CD4^+^ T cells, M1 and M2 macrophages, and negatively correlated with regulatory T cells and activated DCs. Seven immune checkpoints, 14 immune checkpoints and 32 immune factors were significantly up-regulated in the high-risk group, and two immune checkpoints and three immune factors were significantly up-regulated in the low-risk group, and the risk score predicted the effect of immunotherapy. Research findings show genes associated with tricarboxylic acid cycle function and glycolysis in the high-risk group (90). Tumor aerobic glycolysis can lead to nutrient deprivation in the microenvironment, inducing hypoxia with extracellular lactate accumulation, resulting in a suppressive immune microenvironment (91). Therefore, risk models based on (IP6K2 and PLA2G2F) can effectively predict patient prognosis and immunotherapy response and guide individualized immunotherapy (92).

#### 4.3.2 Tryptophan metabolism

Aberrant regulation of tryptophan metabolism is closely associated with the onset and progression of BC (93). Six tryptophan metabolism and bladder cancer immune-related genes (TDO2, ACAT1, IDO1, KMO, KYNU and NAMPT) were identified. Risk models for tryptophan metabolism and immune-related genes including NAMPT, IDO1 and ACAT1 were constructed using Cox regression analysis. Patients were divided into high-risk and low-risk groups. The prognosis of patients in the high-risk group was usually worse than that of patients in the low-risk group. In addition, the 1, 3, and 5 year AUCs of this model were greater than or equal to 0.6. Three types of immune cells (CD8^+^T cells, M1 macrophages, and Treg cells) were found, which were differentially expressed between the two risk subgroups. Compared with the low-risk group, the high-risk group had higher rejection scores but significantly lower dysfunction scores, suggesting that the high-risk group was more likely to have T cell depletion than the low-risk group, while the low-risk group was more likely to have immune cell dysfunction. This risk model can effectively predict the prognosis and immunotherapy response of patients and can guide individualized immunotherapy (94).

### 4.4 Other genes

#### 4.4.1 Autophagy-related genes (ARGs)

Autophagy is considered important for maintaining mitochondrial function and energetic homeostasis in cancer cells. Autophagy plays a crucial role in TME regulation leading to tumor cell migration and invasion, tumor stem cell maintenance and treatment resistance (95). Five prognostic ARGs were found to be significantly associated with OS in bladder cancer patients. Among them, APOL1 was a protective gene, and DIRAS3, NAMPT, P4HB and SPHK1 were considered high-risk genes for poor prognosis in BC. A prognostic risk model based on 5 ARGs was constructed with an AUC value of 0.724. APOL1 was down-regulated in patients with high-risk scores, indicating a good prognosis, while the other 4 risk genes were up-regulated in patients with high-risk scores for a poor prognosis. The correlation between risk score and TME revealed that neutrophil and macrophage M0 and M2 increased with increasing risk score, which correlated with poor pathological staging, and poor prognosis. An increase in risk score was associated with a significant decrease in tumor purity. To facilitate the clinical application and predictive accuracy of the model, a systematic prognostic profile was constructed incorporating clinical factors. With increasing systematic risk, bladder cancer patients had a poorer prognosis. The 3 year OS was 49.0% and 5 year OS was 37.6% in the high-risk group, and the 3 year OS was 77.6% and 5 year OS was 69.0% in the low-risk group. The AUC value for systematic prognostic features was 0.791, which was more accurate than the model based on ARGs alone. Therefore, ARGs may be ideal biomarkers for predicting bladder cancer progression and prognosis (96).

#### 4.4.2 Immune cell infiltration-related genes (IRGs)

Li et al. demonstrated the critical role of TME in bladder cancer progression (97). Different immune cell infiltration abundances showed significant heterogeneity in the genome, transcriptome and biological processes of BC. Five shared immune cells associated with bladder cancer prognosis were identified, including cytotoxic cells, CD8^+^ T cells, T helper cells, T follicular helper cells (TFH) and DCs. Subsequently, immune cell infiltration-related gene markers (FPR1, CIITA, KLRC1, TNFRSF6B, and WFIKKN1) were developed based on immune-related genes (IRGs). Survival analysis revealed AUCs of 0.798, 0.748, and 0.656 at 1, 3, and 5 years, respectively. In conjunction with clinical features, a nomogram was constructed to predict 1, 3, and 5 year OS in BC, and the AUC of the nomograms were 0.774, 0.724, and 0.709, respectively. This model provides a prognostic indicator for BC and a potential immunotherapeutic target (98).

**Figure 3:**
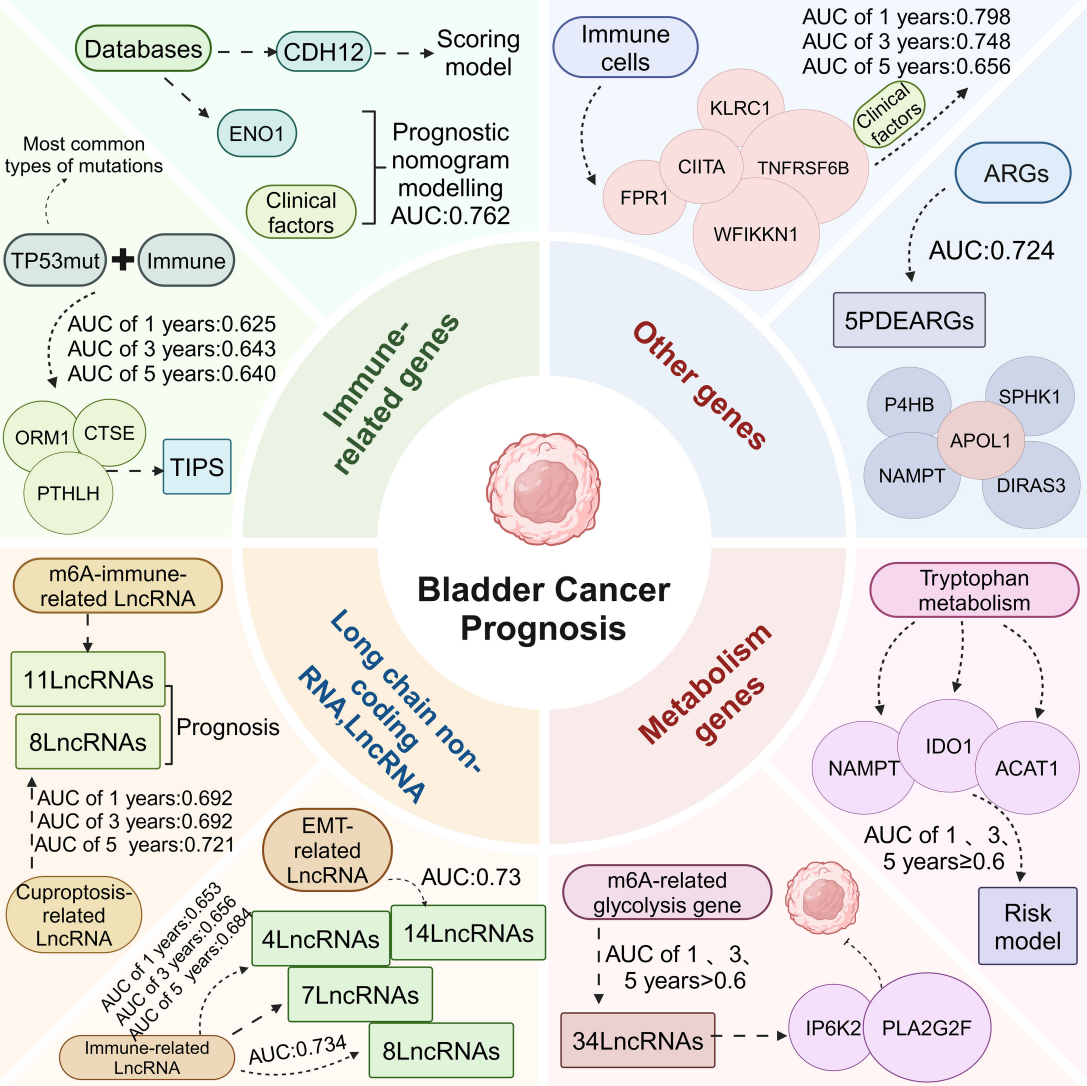
Relationship between immune-related markers and bladder cancer prognosis. Immune-related genes, LncRNA, metabolism genes, and other genes were divided into four parts to construct risk models based on the prognosis of bladder cancer to predict the OS of bladder cancer patients at 1, 3, and 5 years.

In the treatment and prognosis evaluation of BC, four aspects including immune-related genes, LncRNA, metabolic genes, and other factors are closely interconnected, jointly affecting the progression of the disease and treatment outcomes (**Figure 3**). Immune-related genes directly impact the prognosis of BC by participating in the regulation of immune responses and cell interactions within the TME. LncRNA is involved in the regulation of prognosis by modulating the infiltration and function of immune cells, as well as the metabolism and phenotype of tumor cells. m6A-related glycolysis genes and tryptophan metabolism-related genes influence the prognosis of BC by regulating the metabolic state of tumor cells. ARGs and IRGs indirectly affect the prognosis of BC by regulating the composition and function of immune cells in the TME, as well as the autophagy and metabolic state of tumor cells. In summary, these factors interact through different biological pathways and mechanisms, collectively influencing the prognosis of BC. A deeper understanding of the interactions and regulatory networks among these factors can help develop new prognostic evaluation tools and treatment strategies to improve the treatment outcomes for bladder cancer patients.

## 5 Summary and outlook

In the clinical practice of BC, FGFR3 gene mutations are very common, especially in NMIBC. FGFR3 inhibitors have shown anti-tumor cell proliferation and pro-apoptotic effects on bladder cancer cells carrying FGFR3 gene mutations, indicating that the status of FGFR3 gene mutations can predict patients’ response to targeted therapy. The first-generation FGFR inhibitors (anoltonib, ponatinib, dovitinib, lucitanib, lenvatinib, nintedanib) are multi-target inhibitors (99). Due to the reduction in FGFR activity and the increase in side effects, new-generation FGFR inhibitors have made new progress, mainly including erdafitinib for the treatment of BC containing FGFR2-3 mutations (100); infigratinib to evaluate whether it can be used as adjuvant therapy after surgery (101). Rogaratinib has comparable efficacy to standard chemotherapy in patients with FGFR-positive locally advanced or metastatic BC (102). Vofatamab combined with PD-1 inhibitor pembrolizumab significantly enhances the inhibitory activity against tumors carrying FGFR3 mutations (103). Therefore, patients carrying different FGFR3 mutants may have different sensitivities to FGFR inhibitors, and it is necessary to choose the appropriate targeted drug. Additionally, through immunohistochemical staining of tumor tissues, the expression levels of immune checkpoints such as PD-L1, TIGIT, and NKG2A can be detected. The expression levels of these markers are closely related to the patient’s response to immunotherapy. Immune gene expression analysis can assess the expression levels of immune-related genes in tumor tissues. This can be achieved through methods such as gene chip technology or spatial transcriptome sequencing (104). By analyzing immune gene expression, one can understand the infiltration of immune cells in the TME and the secretion levels of cytokines, thereby predicting the potential effects of immunotherapy (105). Using single-cell sequencing or gene expression profiling analysis, the status of immune cells in the TME can be assessed. This method helps predict the patient’s response to ICIs (106). Urine-based DNA or RNA detection technologies are gradually becoming a new method for non-invasive diagnosis, capable of detecting specific gene mutations and methylation status associated with BC (107, 108). Due to the relatively high costs of sequencing experimental equipment and data analysis, their widespread application in large-scale studies is limited.

Immune-related bladder cancer markers play an important role in diagnostic, therapeutic and prognostic assessment and are mainly used to overcome the limitations of current diagnostic, predictive and prognostic tools. They are used to detect BC in asymptomatic or hematuric individuals and as an adjunct to cystoscopy for disease surveillance (109, 110). They also play a potential role as predictors of disease recurrence, progression and survival in patients with non-invasive cancer and in those with advanced disease. The discovery of these markers has provided new insights for early diagnosis, individualized treatment and monitoring of disease progression. Due to the heterogeneity of BC and the requirements of precision medicine, urine cytology combined with cystoscopy is still the gold standard for BC diagnosis in clinical practice, despite the fact that immune markers can diagnose bladder cancer, detect immunotherapy, and predict recurrence. And the potential of a single marker to adequately characterize cancer is still controversial. In the treatment and prognosis evaluation of BC, prognostic feature models constructed based on immune-related markers have shown potential. For instance, the combination of NKG2A and PD-L1 is beneficial for predicting the response of MIBC to treatment. TIGIT and PD-1 classify bladder cancer patients, aiding in providing personalized treatment. Patients with low TIGIT and low PD-1 have a high mutation rate of FGFR3 and are sensitive to FGFR3 targeted therapy. In BC, the expression levels of PD-L1/PD-1 are related to the subtype of the tumor. The expression of PD-L1 is closely related to the grading, staging, and recurrence of BC (111). Moreover, the intensity of PD-L1 expression increases with the progression of BC staging (112), and the expression of PD-1 and PD-L1 in high-grade bladder cancer is higher than in low-grade bladder cancer. The expression level of PD-L1 can serve as an important indicator for predicting the response to immunotherapy, and for patients with high PD-L1 expression, the use of PD-1/PD-L1 blockers may be considered as a priority for treatment (113, 114). However, their clinical application still faces some challenges and limitations. The expression level of PD-L1 as a biomarker for predicting the response to immunotherapy is not completely reliable, and some PD-L1 negative patients may also benefit from immunotherapy. In addition, the unified standards for models predicting the prognosis of BC based on different immune-related markers also need further resolution. Therefore, future research needs to further validate the clinical value of these markers and explore cost-effective, more accurate detection methods, and evaluation standards. In addition, combining immunotherapeutic strategies such as immune checkpoint inhibitors with immune markers may become an important direction for bladder cancer treatment in the future. Ultimately, through in-depth study of immune-related markers, we are expected to achieve the goal of individualized treatment of BC and improve the survival rate and quality of life of patients.

## Glossary

BC: bladder cancer
NMIBC: non-muscle invasive bladder cancer
TURBT: transurethral resection of bladder tumor
BCG: bacille calmette-guérin
MIBC: muscle-invasive bladder cancer
RC: radical cystectomy
TME: tumor microenvironment
ICIs: immune checkpoint inhibitors
TMB: tumor mutation burden
TCGA: the cancer genome atlas
GTEx: genotype tissue expression
OS: overall survival
DSS: disease-specific survival
PFI: progression-free interval
DCs: dendritic cells
NK: natural killer
ssGSEA: single-sample gene set enrichment analysis
ECM: extracellular matrix
COL6A1: collagen VI
NXPH4: neurexophilin 4
m6A: n6-methyladenosine
CNV: copy number variation
TLS: tertiary lymphoid structure
CAFs: cancer-associated fibroblasts
EMT: epithelial-mesenchymal transition
MDSCs: marrow-derived sexual inhibition cells
TILs: tumor-infiltrating lymphocytes
TAMs: tumor-associated macrophages
Treg: regulator T
ACT: adjuvant chemotherapy
TMERS: tumor microenvironment related signals
CDH12: cadherin 12
ICT: immune checkpoint treatment
TIPS: TP53-associated immune prognostic signature
LncRNA: long chain non-coding RNA
IRPLS: immune-related prognostic LncRNA signature
ARGs: autophagy related genes
TFH: T follicular helper cells
IRGs: immune-related genes
